# Factor V deficiency with a unique genetic mutation presenting as post-circumcision bleeding in a neonate, A-case-report

**DOI:** 10.1016/j.amsu.2022.103723

**Published:** 2022-05-05

**Authors:** Fajr M A Sarhan, Ameer Al-Jasim, Raghad H.M. Alwahsh, Islam I.A. Mansour

**Affiliations:** a Al-Quds University, School of Medicine, East Jerusalem, West Bank, Palestine; b University of Baghdad, School of Medicine, Baghdad, Iraq

**Keywords:** Factor V Deficiency, Circumcision, Intracranial hemorrhage, Case report, Literature review

## Abstract

**Introduction and importance:**

Factor V deficiency is a rare bleeding disorder with varying presentations from minor mucosal bleeding to a life-threatening postoperative bleed. Currently, treatment is mainly supportive with Fresh Frozen Plasma.

**Case presentation:**

A previously healthy 14-day-old male presented with an uncontrollable bleeding following a circumcision. Physical examination was normal. Investigations showed hemoglobin 15.5 g/dl, platelets 409000, Prothrombin Time 57 seconds, Partial-Thromboplastin-Time 120 seconds. Mixing study corrected the coagulation profile, and the factor assay showed factor V activity of 11%. Genetic testing showed a pathogenic frameshift mutation in the *F5 gene p.(P9*27Lfs*7) causing premature termination after 7 codons thus the diagnosis of Factor V deficiency was made.

**Clinical discussion:**

In this case, factor V deficiency presented as post-circumcision bleeding. For diagnosis, increased PT and PTT with normal thrombin time increases the index of suspicion for a bleeding disorder. Further testing with coagulation factors assays is required to make the final diagnosis. Factor V deficient patients undergoing surgery should be adequately prepared, and factor V activity level should be maintained at least at 25% of the normal activity level. The patient level prior to the circumcision was unknown, which led to the life threatening bleed.

**Conclusions:**

One of the early presentations of factor V deficiency is a post-circumcision bleeding. Adequate preparation with laboratory tests before circumcision is therefore recommended, especially for high-risk individuals. More than 100 genetic mutations were detected; frameshift mutation involving F5 gene p.(P927Lfs*7) was seen in our case.

## Highlights

• Factor V deficiency is a rare, but a fatal diseasewithout proper diagnosis and management, and can present early as a post-circumcision bleeding.
• Adequate preparation with laboratory tests before circumcision is to be recommended, especially for the products of consanguineous marriage.
• The age at which factor V deficiency presents is variable depending on the degree of the activity of that factor.
• Management for this disorder is complex and needs further investigations for other solutions. Our case's treatment focuses on providing Fresh Frozen Plasma.

## 1 Introduction

Factor V deficiency is a rare bleeding disorder which can be inherited or acquired [1]. The inherited form has an autosomal recessive pattern with an estimated prevalence of 1/1,000,000. On the other hand, the acquired form is caused by inhibitors such as antibodies against factor V which is 10 times less prevalent than the inherited form [2,3].

Regarding the presentation of such a rare condition, it ranges from a minor mucosal bleeding to an extensive postoperative bleeding [1,4].

As for some other types of factor deficiencies, treatment focuses on correcting the factor levels with fresh frozen plasma (FFP) for the inherited form, while plasmapheresis is to be used for the acquired form [1,5]. In this case we present a neonate with an inherited factor V deficiency with a unique genetic mutation that initially presented as an extensive bleeding following a circumcision performed at local settings without adequate preparation.

This paper has been reported in-line with the SCARE 2020 criteria [6].

## 2 Case presentation

A previously healthy 14-day-old male presented with an uncontrollable bleeding following a circumcision. The circumcision, which is a norm in the patient's family, was performed at local settings by his primary healthcare physician without adequate preparation in terms of laboratory tests, and was complicated by profuse bleeding despite sutures placement. There was no fever, vomiting, diarrhea, shortness of breath or jaundice upon presentation to the emergency room (ER).

This neonate is a product of a normal vaginal delivery of a 29-year-old mother that had undergone all required pregnancy tests which came back normal. Apgar Scores were 8 and 9 at 1 minute and 5 minutes, respectively. Physical examination after the delivery did not show any findings of clinical importance. His first 2 weeks were uneventful. Weight at birth was 3.1 kg.

The patient's blood group is AB+ with an unknown maternal blood group, which makes it relatively more difficult to find matching blood and plasma.

Regarding the family history, the patient is a member of a family of five, with two older brothers who are completely healthy with no history of bleeding tendencies, as are the parents, who are first degree cousins. The patient is not taking any medications, and there is no family history of bleeding episodes. No psychosocial history of clinical importance was reported. The patient doesn't have any known food or drug allergy.

Regarding vital signs at the presentation, the patient's heart rate was 166 beats/minute, respiratory rate was 62 breaths/minute, blood pressure was 82/51 mmHg and the rectal temperature was 37.3 °C.

Initial workup included a Complete Blood Count (CBC) that showed a hemoglobin of 15.5 g/dl, Hematocrit (HCT) of 46%, White Blood Cells count (WBC) of 10.15*109/L, platelets (PLT) count of 409000, Prothrombin time (PT) of 57 seconds, Partial Thromboplastin time (PTT) of 120 seconds, and International normalized Ratio (INR) of 4.69.

Initial management with vitamin K and Fresh Frozen Plasma was made. The patient was stabilized and monitored for the next three days. His first admission lasted six days.

An inpatient follow up with coagulation profile was done on the admission to the pediatric ward and showed elevated PT, PTT, and INR with normal platelets count despite the initial management (Fig. 1A). A presumptive diagnosis of coagulation disorder was made. A mixing study was sent and the results showed a correction in the coagulation profile, which confirmed a factor deficiency. Factors assay showed normal results for all factors except factor V which was only 11% active. Factor V's normal activity range is between 50% and 200%.Fig. 1B shows the activity level of factor V during the first admission.

**Fig. 1A:**
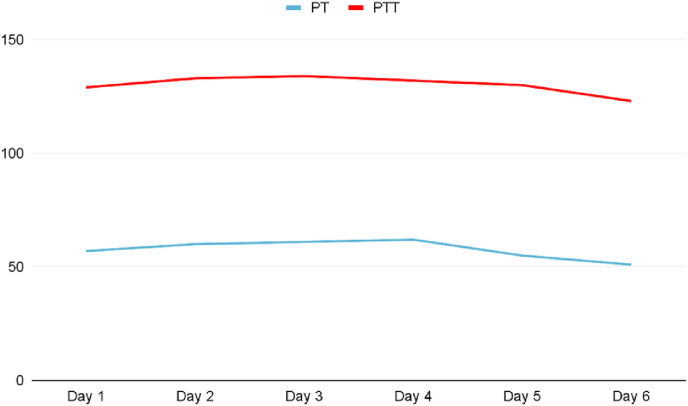
PT and PTT

**Fig. 1B:**
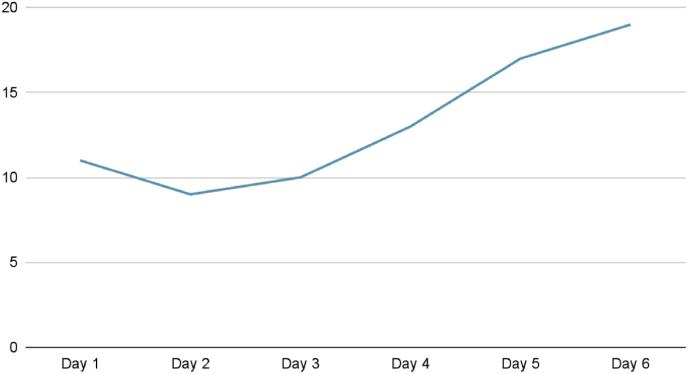
First admission Factor V levels.

The patient was discharged with the diagnosis of factor V deficiency, and the family were advised to follow up at the hematology clinic. Genetic testing was ordered for the patient, and the parents.

Two weeks later, the patient was brought to the emergency room by his mother complaining of seizures that she described as alternating eye movements and bilateral arm jerking. The patient's initial workup showed elevated PT, PTT (Fig. 2A) and low factor V activity (Fig. 2B). Urgent head CT scan was done and showed intracranial hemorrhage (Fig. 3).

**Fig. 2A:**
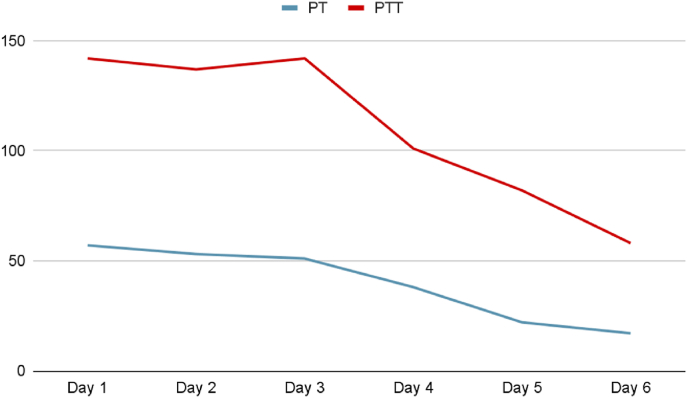
PT and PTT at the second admission.

**Fig. 2B:**
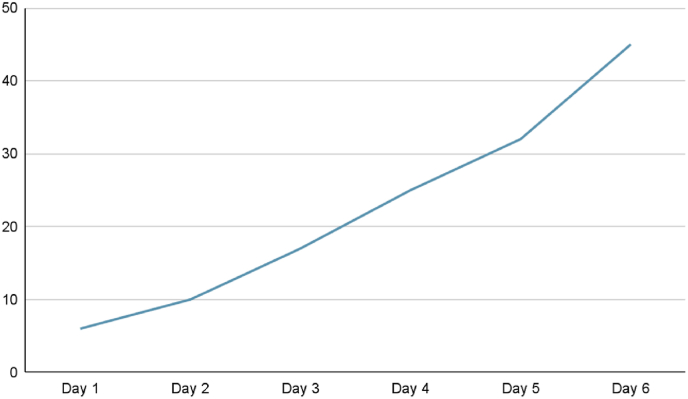
Factor V levels at the second admission.

**Fig. 3:**
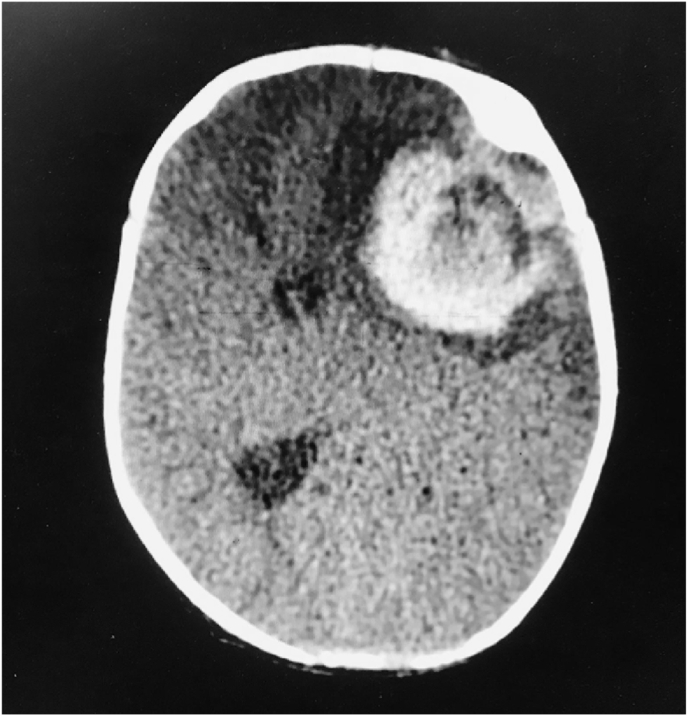
Head Computed Tomography scan demonstrates an acute hemorrhage in the left frontal lobe with associated vasogenic edema and severe left-to-right midline shift.

The patient was started on Phenytoin, Mannitol, and the neurosurgery team was consulted, who decided that craniotomy and evacuation are required to stabilize the patient's condition. Fresh Frozen Plasma and vitamin K were given prior to and after the surgery.

After 9 days of admission, no complaints or complications were noted, and the patient is now discharged and is being followed in the outpatient hematology clinic.

6 weeks later, genetic testing results showed a homozygous pathogenic frameshift mutation in the *F5 gene* -*p.(P9*27Lfs*7) gene-causing premature termination after 7 codons. Both parents were heterozygous for this mutation.

## 3 Clinical discussion

Factor V Deficiency (Owren's Disease or parahemophilia) is a rare bleeding disorder that presents as an extensive bleeding from the mucosal membranes or after surgical procedures [1,2]. Factor V is an essential component of the combined pathway of the coagulation cascade. It has a pivotal role as a cofactor for the conversion of factor II (prothrombin) into factor IIa (thrombin) via factor X [3]. If the factor V is deficient, thrombin will be missing and the fibrin mesh will not form. Factor V is regulated by Protein C, which helps maintain the coagulation cascade by degrading Factor V and VIII [3,4].

Factor V deficiency has two etiologies, Inherited and acquired. The mode of inheritance is autosomal recessive. Factor V gene is encoded on the long arm of chromosome 1 [4]. Each of those genes is inherited from a carrier parent, and the presence of the two defective genes is required for the disease to manifest, thus, those who are products of consanguineous marriage are at risk acquiring it [5]. 100 mutations at least, have been detected in the gene, causing a decreased production or a malfunctioning protein [7]. Our case showed a pathogenic frameshift mutation in the *F5 gene p.(P9*27Lfs*7) gene causing a premature termination after codon 7 leading to a decreased production of Factor V. It can present after the first surgical procedure, as in our case which presented as a post-circumcision bleed. The acquired form of the disease is caused by the formation of antibodies against Factor V [8]. Risk factors for the acquired form include: previous surgical procedures, blood transfusions, antibiotics [1,2]. Acquired deficiency can present at any age with mixed presentations which makes it difficult to be diagnosed [9].

For diagnosis, increased PT and PTT with normal thrombin time increases the index of suspicion for such a rare bleeding disorder. This increase in PT and PTT is due to the presence of factor V in the combined pathway. Mixing studies with normal plasma will correct the coagulation profile [10]. Further testing with specific factors assays is required to make the diagnosis [1].

An Article regarding factor V deficiency stated that platelets transfusion can benefit the bleeding patients more than the traditional usage of Fresh Frozen Plasma (FFP), as factor V that is stored in the alpha granules of the platelets makes it less prone to degradation, and it will be released when needed, specifically at the sites of vascular injuries. If platelets are not available, FFP is to be administered [8].

Elimination of the factor inhibitors should be considered in patients with minor bleeding due to acquired factor V deficiency. Clinical correlation must be considered for patients with a history of thrombosis [8,11].

Gene therapy is showing a promising step in treating this disease. Nakamura et al. in their recent article showed that the correction of the factor deficiency can be made using pluripotent stem cells, which restores the normal activity level of Factor V [12].

Factor V deficient patients who are undergoing surgery should be adequately prepared and the activity level of factor V should be maintained at 25% of normal as a lower cutoff point [13]. This level was not achieved in our patient prior to his circumcision, as well as prior to the brain evacuation surgery in the second admission due to the life threatening emergency.

During the postoperative period, patients should receive fresh frozen plasma at a rate of 15 ml/kg/dose twice a day for 10 days then once daily for another 10 days [14].

## 4 Conclusions and take home messages

This case illustrates that post-circumcision bleeding can be the presenting feature of factor V deficiency earlier in life. Adequate preparation with laboratory tests before circumcision is therefore recommended especially in high risk individuals as in those with family history of inherited bleeding disorders or products of consanguineous marriage.

The age at which factor V deficiency presents varies depending on the factor's activity level. This case highlights its presentation in the neonatal period with a unique genetic mutation, among other 100 mutations, involving the F5 gene -p.(P927Lfs*7) gene-.

## Ethical approval

N/A.

## Sources of funding

N/A.

## Author contribution

Writing the manuscript: Fajr M A Sarhan, Ameer Al-Jasim, Raghad H. M. Alwahsh, Islam I. A. Mansour, Designing the figures: Fajr M A Sarhan, Islam I. A. Mansour, Raghd H. M. Alwahsh, Reviewing & editing the manuscript: Fajr M A Sarhan, Ameer Al-Jasim

## Consent

Written informed consent was obtained from the patient's mother for Publication of this case report and accompanying image. A copy of the informed consent is available for review by the Editor-in-Chief of this journal on request.

## Research registration

● Name of the registry: N/A.
● Unique Identifying number or registration ID: N/A
● Hyperlink to your specific registration (must be publicly accessible and will be checked): N/A

## Guarantor

Fajr M A Sarhan, M.D. Al-Quds University, Ramallah-West Bank, Palestine. fajr.sarhan@students.alquds.edu

## Provenance and peer review

Not commissioned, externally peer-reviewed.

## Declaration of competing interest

The authors declare no conflict of interest.
